# Some Aspects of the Criminal

**Published:** 1908-04-11

**Authors:** 


### Some Aspects of the Criminal.

Perhaps the time is hardly yet ripe for crime to be looked upon in the light of a disease. There are too many indignant moralists about who consider that the reward of their own virtue is the satisfactory condemnation of the vicious; and nothing will persuade them that the criminal is not responsible for his wrong-doing, because they believe that to themselves alone they owe their own virtue. So far as the practical treatment of crime is concerned, the methods that now obtain will have to be continued, not because they are entirely satisfactory, but simply for want of agreement about better. But this will not prevent the endeavour to apply gradually a treatment based on scientific reasoning. Our present treatment is, perforce, a rather pitiful compromise. For example, one school demands that criminals condemned to death or to long sentences be handed over to the medical faculty for purposes of physiological or psychological experiment. To kill a man right out or put him away for a long period, in which he performs absurd and scarcely useful duties, is, on this view, pure waste of opportunity. Humanitarians also demand the abolition of capital punishment, though on quite different grounds. On the other hand, lawyers believe that penalties, especially the death penalty, deter many from crime who might otherwise commit it. As far as can be judged, the tendency nowadays is almost universally towards a practical utilitarianism, and it is regrettable that in the matter of the criminal so little consistency is shown.

Before any advance can be made, it is necessary to realise the essential characteristics of the criminal from a non-moral point of view. The criminal approaches the animal rather strikingly. He is essentially without self-introspection and consciousness of self; he is a fatalist whose acts seem compelled by some inward *trieb* or impulse, and in action he finds his end. Freedom is to him not only impossible, but hateful, and he is consequently against all laws, which are, of course, the basis of freedom as opposed to license. The frequency of epilepsy among criminals may be regarded in many cases both as cause and effect of his condition. These tendencies in the individual do not inevitably result in criminal practices. The point which we would emphasise, however, is this: that society expresses, roughly, the consent of a majority of individuals to a certain manner of life. Against those who are in open and unceasing opposition to this manner society is bound to protect itself, and the means used should be as scientific as those employed in the case of grave diseases. The more conspicuous features of the character of the criminal being recognised, it should be the endeavour of medical science to suggest a remedy. Despite the many who would urge that environment is of paramount importance in the matter, we would suggest that it is not so important as origin. The habitual criminal—and such a one is not difficult to distinguish—should be prevented from reproducing his species. There are well-known and not infrequent cases of boys who, at the age of twenty, have had ten or fifteen convictions. Such youthful offenders should not be incarcerated in the usual slipshod way: the only form of profit they are ever likely to yield the State is as material for scientific study, and they should at all costs be deprived of the chance of perpetuating their characteristics. The justification for such a policy is to be found both in society and nature. Might is, *ipso facto*, right. Gloss we this fact never so pleasantly, the existence of the policeman proves it to us daily.

